# Upregulation of calprotectin in mild IgE-mediated ovalbumin hypersensitivity

**DOI:** 10.18632/oncotarget.16954

**Published:** 2017-04-08

**Authors:** Qingling Zhu, Feng Li, Junli Wang, Jingqiu Ma, Xiaoyang Sheng

**Affiliations:** ^1^ Department of Child and Adolescent Healthcare, MOE-Shanghai Key Laboratory of Children's Environmental Health, Xinhua Hospital Affiliated to Shanghai Jiao Tong University School of Medicine, 200092, Shanghai, China

**Keywords:** calprotectin, S100A8/A9, food hypersensitivity, IgE, animal experiment

## Abstract

Calprotectin, also known as S100A8/A9, has been linked to gut inflammation caused by IgE-mediated food hypersensitivities, but the pathophysiologic abnormalities it causes remain to be determined. We created a mild food hypersensitivity model through oral gavage of ovalbumin in Norway brown rats without using immune adjuvant. Changes in the levels of calprotectin and inflammation-associated cytokines were then observed over time. We found that fecal calprotectin as well as jejunal and liver TLR4, TNF-α, NF-κB, IL-1β, and IL-6 were upregulated in hypersensitive rats. Additionally, the influence of calprotectin on CD4^+^ T and dendritic cells was observed by co-culturing CD4^+^ T cells with dendritic cells, which revealed a shift toward increased Th2 T cells in calprotectin-treated cultures. These results suggest that calprotectin, along with other inflammatory factors, promotes the inflammation seen in mild food allergy.

## INTRODUCTION

Food allergy, one of the most prevalent ailments in young children, is defined as an adverse health effect caused by a specific immune response that takes place reproducibly upon exposure to a given food [1]. Because both immune and non-immune functions in children, especially infants, are immature, upon consumption of an offending food antigen they are more vulnerable to allergic reactions with varying degrees of severity. In the developed countries, an estimated 5%–10% of children suffer from food allergy, mainly caused by allergen-specific IgE [2–4]. New evidence shows that food allergy, especially in young children [2], is on the rise and remains a major public health problem with no curative therapy [1]. A better understanding of IgE-mediated food allergy and active study for effective prevention or treatment is essential.

Calprotectin, also known as S100A8/A9, MRP8/14, and calgranulin A/B, is a 36 kDa calcium and zinc binding protein, which belongs to the S100 family [5]. S100A8 and S100A9 create a heterologous dimer, S100A8/A9, in a Ca^2+^ dependent manner. S100A8/A9 is widely distributed in human cells, tissues, and body fluids and constitutes the main protein of neutrophils and macrophages, making it an important part of the innate immune system [6–8]. The heterodimer inhibits microbial activity (including bacteria, fungi) and plays a role in proliferation, immune regulation, inhibition of matrix metalloproteinase activity and other biological functions [5, 9]. In addition, S100A8/A9 is a neutrophilic chemotactic factor, the level of which will increase 5–40 fold above normal in the plasma in response to diseases affecting neutrophil activity [10]. Fecal calprotectin, an applicable surrogate marker for gut mucosal inflammation [11] has received attention due to its altered level in both children [12–14] and adults [15] with food allergy. In 2010, fecal calprotectin was demonstrated to predict food allergy [14]. In 2011, Waligora-Dupriet et al. [13] found that the level of fecal calprotectin in infants with food allergy was twice that of infants without food allergy. Beser et al. [12] obtained similar results when they compared infants with milk allergy to healthy infants.

The objective of this study was to define the role of calprotectin activity on the sensitization process and allergic reaction in IgE-mediated food allergy, providing more laboratory and basic data for food allergy by using a widely used Brown Norway (BN) rat model of oral food allergy, a high immunoglobulin (especially IgE) breed [16–18].

## RESULTS

### Hypersensitive rats do not exhibit any changes in body weight or length

**Figure 1:**
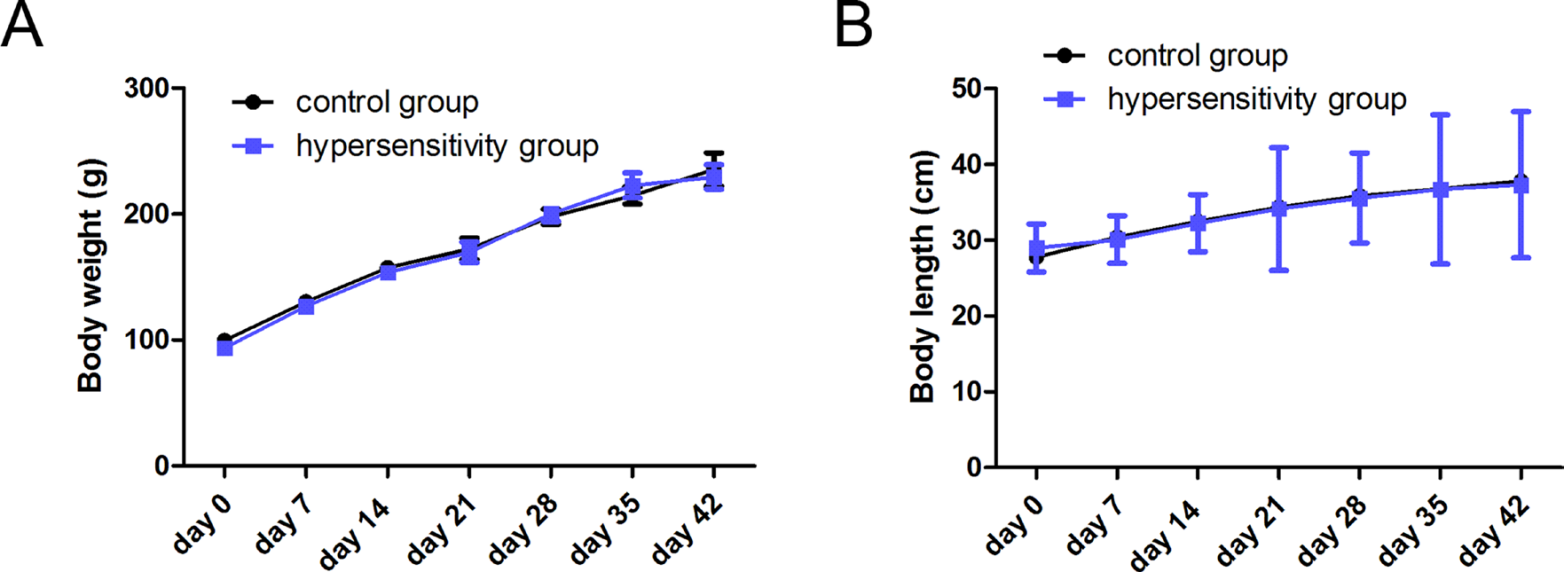
Body weight and length Body weight (**A**) and length (**B**) were monitored weekly from the beginning of the study to the end. There was no significant difference between two groups from days 7 to days 42 (day 7 to 35, *n* = 5/group; day 42, *n* = 15/group, *P* all > 0.05).

There was no accidental death or other loss of experimental rats during the course developing and testing the hypersensitivity model. All rats were analyzed. All experimental rats were 3-week-old males (*n* = 80). The baseline data of rats’ weight and length between the two groups were comparable (*P* > 0.05) (Table 1). Body weight and length were monitored throughout the study and were similar between the two groups from day 7 to 42 (Figure 1A and 1B) (*P* all > 0.05).

**Table 1:** The baseline of control group and hypersensitivity group (*n* = 40, $\bar{x}$ ± s, g, cm)

	Weight	Length
Control group	99.71 ± 3.57	27.76 ± 0.46
Hypersensitivity group	93.62 ± 3.16	28.98 ± 1.68
*P* value	> 0.05	> 0.05

### Evaluation of animal models of food allergy

**Figure 2:**
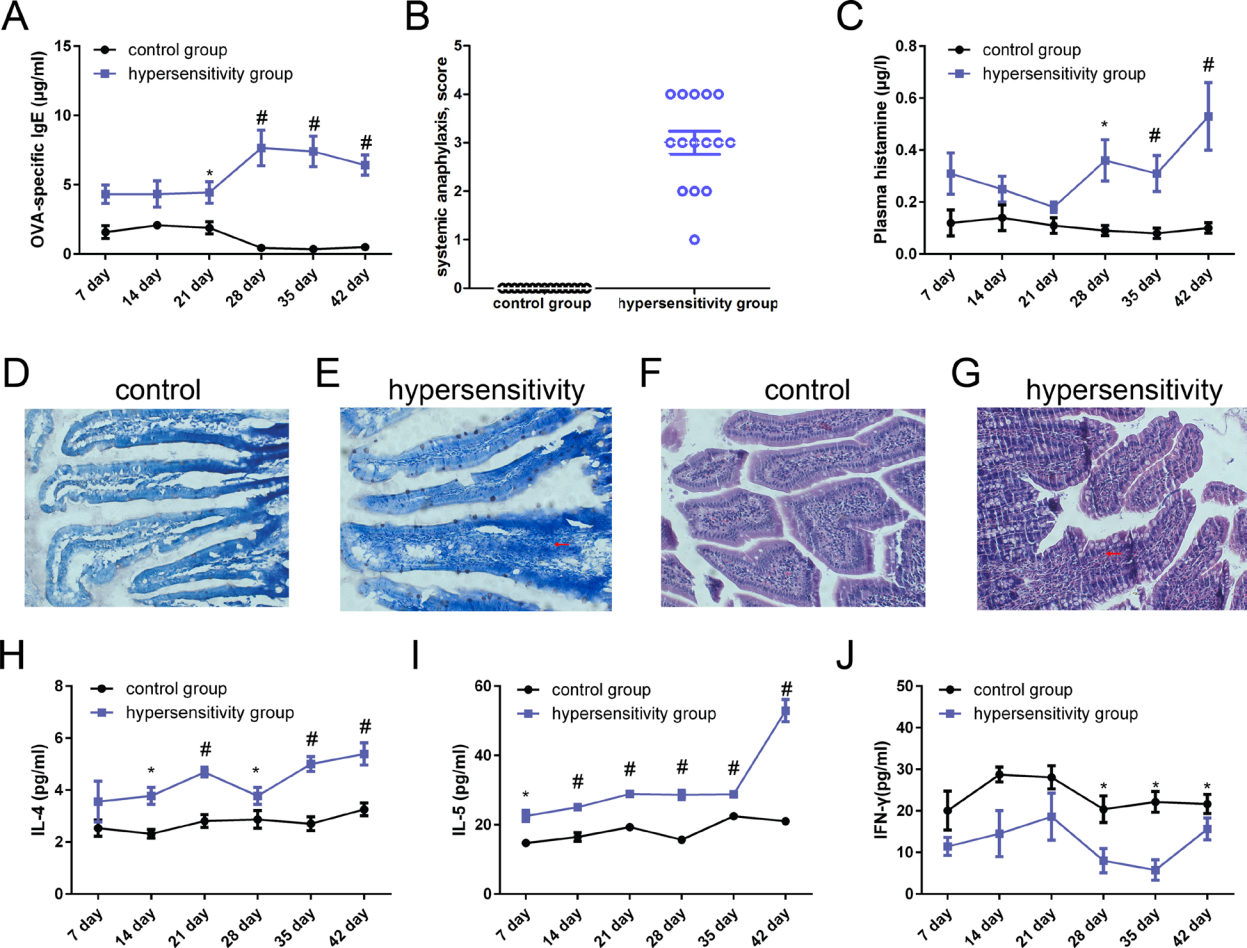
Evaluation of animal models of food allergy Blood and tissues from hypersensitivity group and control group of rats (day 7 to 35, *n* = 5/group; day 42, *n* = 15/group) were obtained weekly after sensitization and final challenge. (**A**) Levels of ovalbumin-specific IgE. Ovalbumin-specific IgE concentrations were detected by ELISA. Data are given as means ± SEM. No immune adjuvant was used. (**B**) Ovalbumin antigen-induced systemic anaphylaxis analysis. On day 42 (*n* = 15/group), each rat in the hypersensitivity group was challenged by means of intragastric gavage with 100 mg ovalbumin. Rats in the control group were false-challenged by normal saline of the same quantity. Anaphylactic symptoms were evaluated 40 minutes after high dose ovalbumin challenge or false-challenge, and a scoring system was used. (**C**) Plasma histamine levels. Histamine levels were determined by using a commercial enzyme immunoassay kit. (**D** and **E**) Antigen-driven mast cell activation in rats’ jejunum, ×200. A representative section showing toluidine blue staining on jejunum tissue: D, Control group; E, hypersensitivity group. Mast cells are stained a deep blue color. There were numerous mast cells in jejunal mucosa, cytoplasmic granules were purple, the nucleus was blue, and there were nuclei with irregular oval or lobulated shape. Cell membranes were ruptured and the nuclei were oblong to discharge purple granules. (**F** and **G**) Antigen-driven eosinophils in rats’ jejunum. A representative section showing HE staining on jejunum tissues, (F) Control group; G, hypersensitivity group. Eosinophils were stained a deep purple color. Eosinophils were round or oval, the cytoplasm was full of red eosinophils stained by HE, and the nucleus was mishapen with visible pertrusions. Some eosinophils were broken with particles scattered around the cell; (**H**, **I**, and **J**) Th2-associated and Th1-associated cytokine profile. H, I and J were the levels of IL-4, IL-5 and IFN-γ respectively in spleen (day 7 to 35, *n* = 5/group; day 42, *n* = 15/group). *P < 0.05 ; ^#^*P* < 0.01 compared with control group.

To trace the variations in antigen-specific IgE, we measured IgE concentrations weekly after administration of oral ovalbumin and compared responses elicited in the hypersensitivity and control groups. Ovalbumin-specific IgE concentrations increased distinctly from day 7 to 42 in the hypersensitivity group (Figure 2A). Statistically significant differences were found on days 21 to 42 between the two groups (*P* all < 0.05). According to the allergic decision criteria mentioned in the methods section, three rats (3/5, 60%) on day 7, three rats (3/5, 60%) on day 14, three rats (3/5, 60%) on day 21, four rats (4/5, 80%) on day 28, four rats (4/5, 80%) on day 35, and twelve rats (12/15, 80%) on day 42 were allergic.

On day 42, rats were fed with 100 mg of ovalbumin by intragastric gavage, and anaphylactic symptom scoring was used to evaluate within 30 to 40 minutes. In the hypersensitivity group one rat presented with a symptom score of 1, three rats reached a score of 2, six rats reached a score of 3, and five rats reached a score of 4; none reached a score of 5. Rats in the control group did not show any allergic symptoms, which was marked as a 0 score. Rats in the hypersensitivity group (total score, 45) exhibited more severe reactions than rats in the control group (total score, 0) (Figure 2B).

Plasma histamine levels were obviously elevated in the hypersensitive rats compared to the control group rats. There were statistically significant differences from day 28 to 42 between the two groups, and the level of histamine in the hypersensitivity group peaked on day 42 (Figure 2C) (*P* all < 0.05).

Histologic staining was used to determine the number of mast cells and granulation status in the jejunum. There were numerous mast cells in the jejunal mucosa: cytoplasmic granules were purple, and the nucleus was stained blue. In addition, there were nuclei with irregular oval or lobulated shape. Cell membranes were ruptured, and to discharge purple granules. Mast cells were rarely seen in the control group (Figure 2D), while they were increased and degranulated in the hypersensitivity group (Figure 2E).

Eosinophils were round or oval-shaped. The cytoplasm was full of red eosinophils stained by HE, and the nucleus was mishapen with visible extrusions. Some eosinophils were broken with particles scattered around the cell. As seen in Figure 2F, eosinophilia was rare in the control group, but extended into the intestinal tissue in the hypersensitivity group (Figure 2G).

As shown in Figure 2H, 2I there was an enhanced production of Th2-associated cytokines IL-4 and IL-5 in spleen from day 7 to 42 in the hypersensitivity group, and significant differences were found from day 14 to 42 for IL-4 and from day 7 to 42 for IL-5. The Th1-associated cytokine IFN-γ was expressed at significantly lower levels in the hypersensitivity group from day 28 to 42 (Figure 2J) (*P* all < 0.05).

### The expression of calprotectin

**Figure 3:**
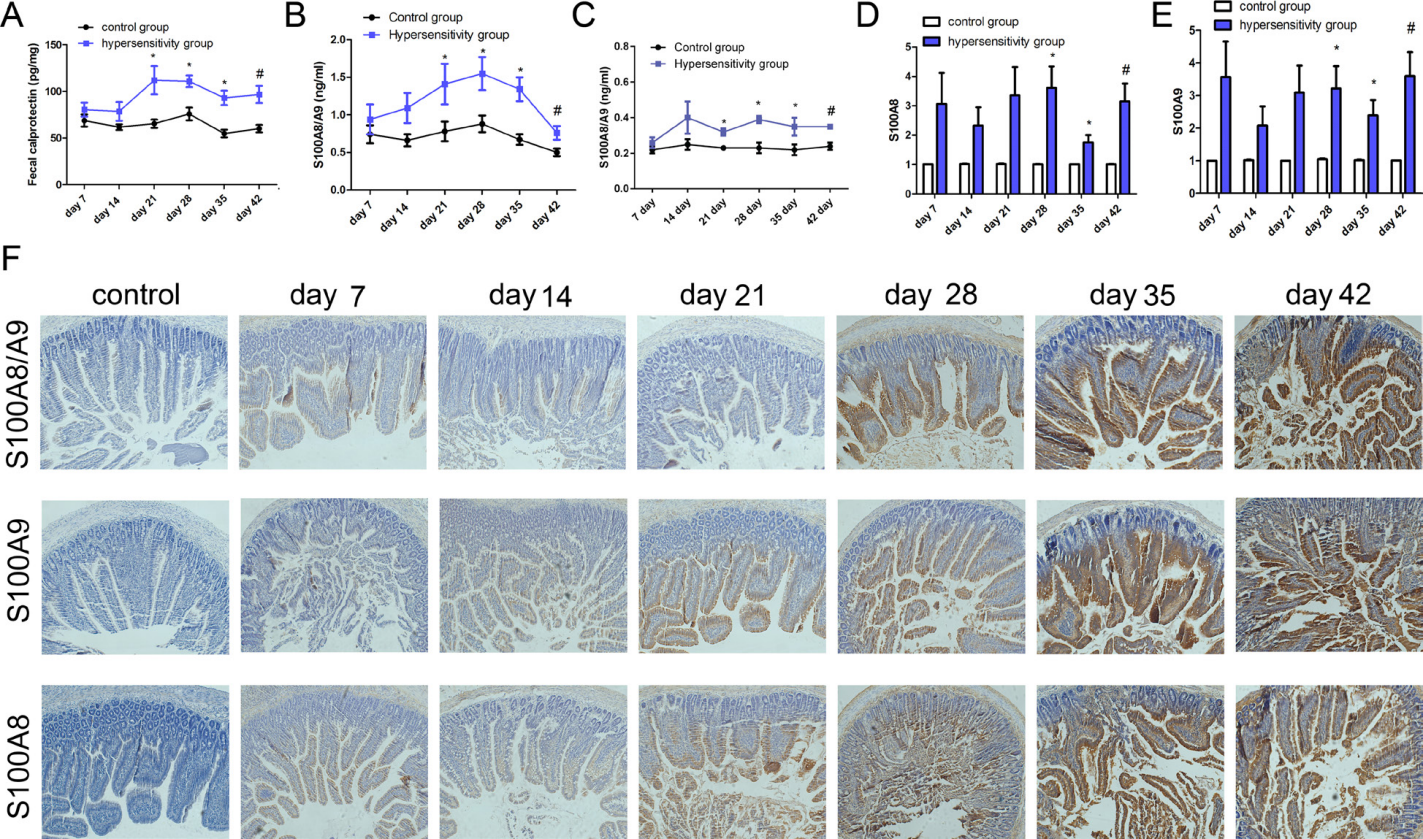
The expression of calprotectin ELISA was used to determine the level of calprotectin in feces and S100A8/A9 in tissue and blood. (**A**) Fecal calprotectin levels. Fecal samples were collected weekly after sensitization or challenge. (**B**) The level of S100A8/A9 in jejunum; (**C**) The level S100A8/A9 in plasma. PCR was used to test the expression of S100A8 and S100A9 in jejunum. (**D** and **E**) were the levels of S100A8 and S100A9 in jejunum; (**F**) Immunohistochemistry was used to detect the expression of S100A8/A9, S100A8, and S100A9 in jejunum. From left to right there were control group, hypersensitivity groups from day 7 to day 42; from up to down, there were the expression of S100A8/A9, S100A9 and S100A8 in jejunum, respectively. *P < 0.05 ; ^#^*P* < 0.01 compared with control group.

To characterize the changes in the level of calprotectin in feces during the sensitization process, feces was collected from each mouse in the two groups weekly after sensitization and analyzed by ELISA. As shown in Figure 3A, there was a remarkable increase in fecal calprotectin after day 14 in the hypersensitivity group; statistically significant differences were observed from day 21 to 42 (*P* all < 0.05).

As presented in Figure 3B, the level of S100A8/A9 in jejunum increased from day 7 to 42, and statistically significant differences between hypersensitivity and control groups were found from day 21 to 42 (*P* all < 0.05). A gentle rise is presented in Figure 3C, showing the changes of S100A8/A9 in plasma with statistically significant differences from day 21 to 42 between the two groups (*P* all < 0.05).

Figure 3D, 3E display the mRNA expression of S100A8 and S100A9 in jejunum, both of which had significantly higher levels from day 28 to 42 in the hypersensitivity group (*P* all < 0.05). The expression of S100A8, S100A9, and S100A8/A9 in jejunum in the hypersensitivity group from day 7 to 42 were obviously increased as shown by immunohistochemistry (Figure 3F).

### Quantification of the inflammation-associated cytokines: TLR4, TNF-α, NF-κB, IL-1β, and IL-6

**Figure 4:**
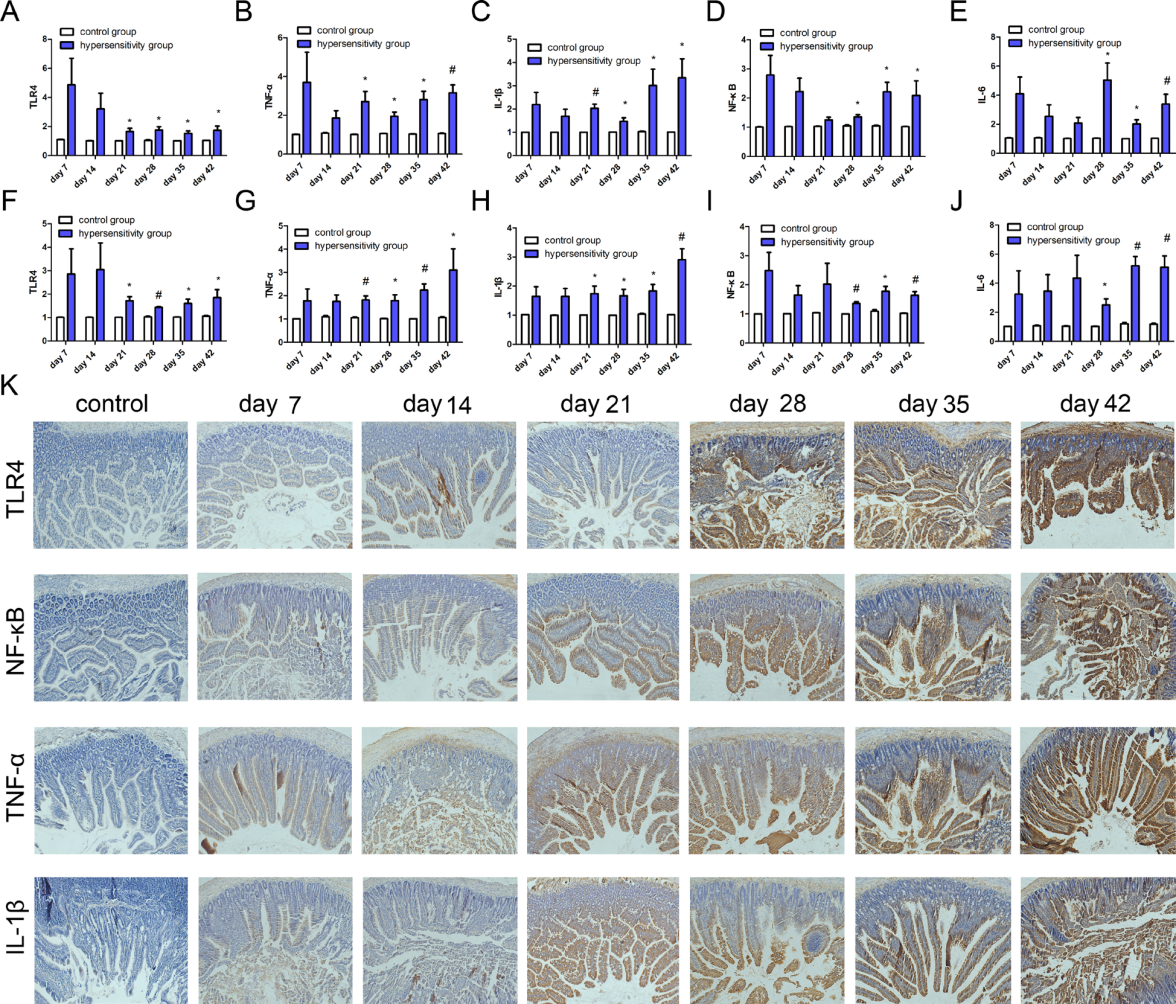
The expression of inflammation-associated cytokines PCR was used to detect the mRNA expression of TLR4, NF-κB, TNF-α, IL-1β, and IL-6. (**A**, **B**, **C**, **D**, and **E**) show the expression of TLR4, NF-κB, TNF-α, IL-1β, and IL-6 in jejunum; (**F**, **G**, **H**, **I** and **J**) show the expression of TLR4, NF-κB, TNF-α, IL-1β, and IL-6 mRNA in liver. *P < 0.05 ; ^#^*P* < 0.01. (**K**) shows the expression of TLR4, NF-κB, TNF-α, and IL-1β. from left to right are control group, hypersensitivity groups from day 7 to day 42; and from top to bottom, expression of TLR4, NF-κB, TNF-α, and IL-1β in jejunum.

We found higher levels of TLR4, TNF-α, NF-κB, IL-1β, and IL-6 mRNA in jejunum in the hypersensitivity group, with statistically significant differences from day 21 to 42 for TLR4, TNF-α, and IL-1β (Figure 4A, 4B and 4C) and from day 28 to 42 for NF-κB and IL-6 (Figure 4D and 4E). In liver, the hypersensitivity group also showed higher levels of these five inflammation-associated cytokines compared to the control group, and statistically significant differences were seen from day 21 to 42 for TLR4, TNF-α, and IL-1β (Figure 4F, 4G and 4H), and from day 28 to 42 for NF-κB and IL-6 (Figure 4I and 4J) (*P* all < 0.05).

The protein expression of TLR4, TNF-α, NF-Κb and IL-1β was also detected by immunohistochemistry. The expression of TLR4, NF-κB, TNF-α and IL-1β in jejunum from day 7 to 42 were highly increased in the hypersensitivity group, but not in the control group (Figure 4K).

### The relationship of fecal calprotectin with S100A8/A9, TLR4, NF-κB or TNF-α

There were positive correlations for fecal calprotectin with plasma S100A8/A9, TLR4, NF-κB and TNF-α by *Spearman correlation* (Spearman's rho = 0.324, *P* = 0.003; Spearman's rho = 0.368, *P* = 0.001; Spearman's rho = 0.382, *P* < 0.001; Spearman's rho = 0.267, *P* = 0.017, respectively).

### The expression of DC surface molecules, IL-4, IL-5, and INF-γ after DC and CD4^+^T cell co-culture

**Figure 5:**
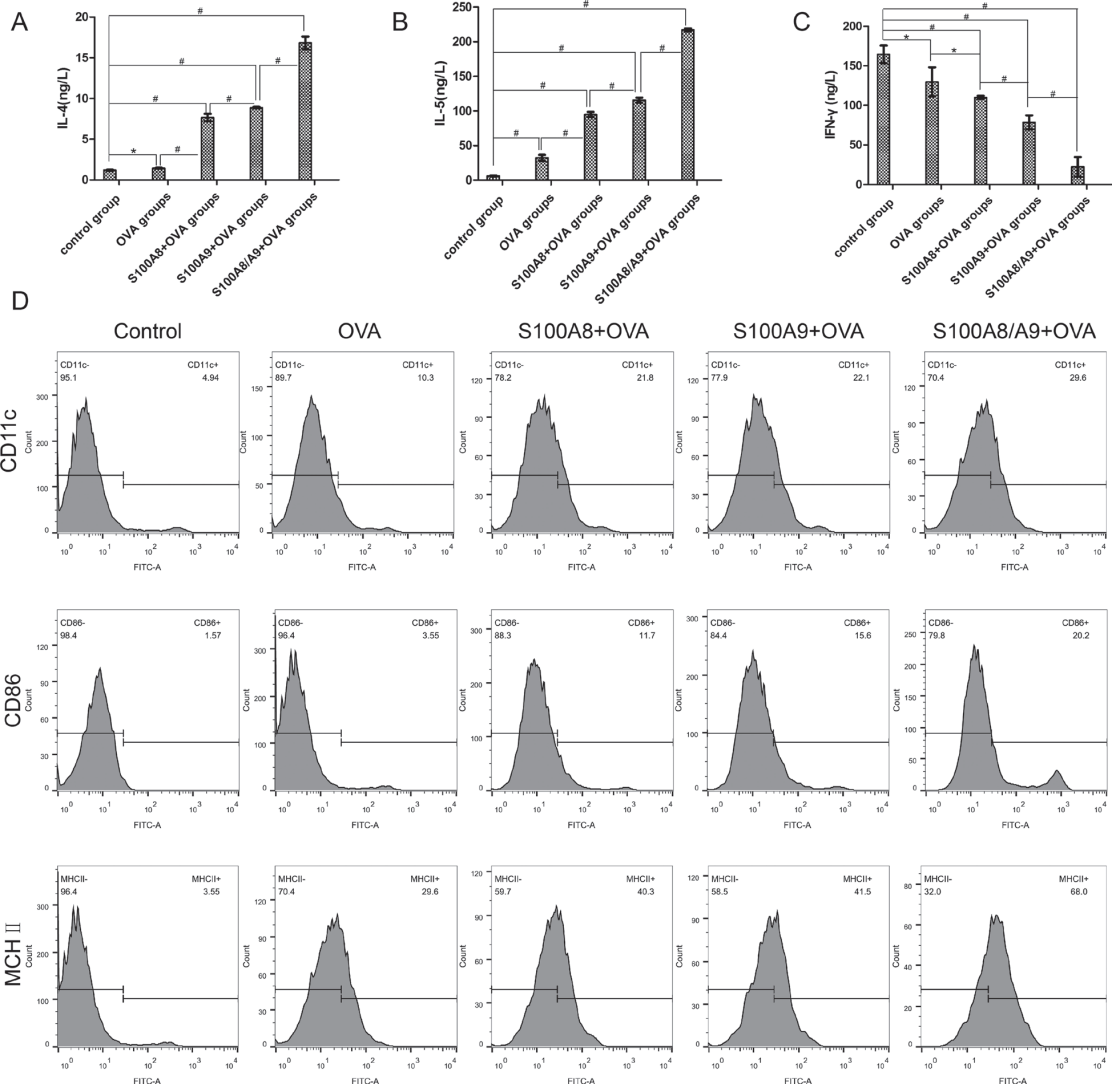
The expression of IL-4, IL-5, INF-γ, and DC surface molecules CD11c, CD86 and MHC II after DC and CD4^+^T cell co-culture ELISA was used to detect the expression of Th1-associated cytokines INF-γ and Th2-associated cytokines IL-4 and IL-5. (**A**, **B** and **C**) are the expression of IL-4, IL-5 and INF-γ in control group, ovalbumin group, S100A8+ovalbumin group, S100A9+ovalbumin group, and S100A8/9+ovalbumin group respectively. *P < 0.05 ; ^#^*P* < 0.01. (**D**) the level of CD11c^+^, CD86^+^, and MHC II^+^ detected by flow cytometry. From left to right are control group, ovalbumin group, S100A8+ovalbumin group, S100A9+ovalbumin group, and S100A8/9+ovalbumin group respectively; from top to bottom, are the expression of CD11c^+^, CD86^+^, and MHC II^+^.

The expression of Th2-associated cytokines IL-4 and IL-5 increased gradually from control group to ovalbumin group, S100A8+ovalbumin group, S100A9+ovalbumin group, and S100A8/A9+ovalbumin group. Significant differences were found between each group (*P* all < 0.05) (Figure 5A, 5B). Conversely, the expression of the Th1-associated cytokine INF-γ gradually decreased from control group to ovalbumin group, S100A8+ovalbumin group, S100A9+ovalbumin group, and S100A8/A9+ovalbumin group (Figure 5C). There were significant differences between each group (*P* all < 0.05).

As shown in Figure 5D, the expression of CD11c^+^ was highest in the combination of S100A8/A9+ovalbumin groups and reduced gradually from the S100A9+ovalbumin groups to S100A8+ovalbumin groups, ovalbumin groups, and control group. The control group had the lowest level. The same trend is also found in CD86^+^ and MHC II^+^ (Figure 5D).

## DISCUSSION

The understanding of the cellular and molecular mechanisms of allergic inflammation pathogenesis has rapidly progressed since 2010 [19]. Clinical research by Berni et al. [20] suggested that children with allergic colitis had an obviously increased level of fecal calprotectin during the active stage, which reduced to normal after four weeks of adjusted diet. Some reported that children suffering from food allergy presented 2-fold higher concentration of fecal calprotectin compared with healthy children [13]. As gastrointestinal symptoms improved or vanished, the level of fecal calprotectin significantly reduced [21]. Huang et al. [22] pointed out that the increased calprotectin in the serum is a potential biomarker of trichloroethylene-induced hypersensitivity dermatitis for clinical diagnosis and drug treatment. Similar views were also presented by Burri [23], and Manz [24], who found calprotectin in feces can improve the diagnosis of abdominal complains. It was also found to be a cost-effective diagnostic tool for diagnosing new-onset inflammatory bowel disease (IBD) [25], as well as the identification of quiescent Crohn's disease [26]. In this study, we generated a BN rat model of anaphylaxis in which IgE-mediated ovalbumin hypersensitivity was induced by oral sensitization and challenge without any immune adjuvant. A proper food allergy model without adjuvant seems advisable because an adjuvant may have an impact on the IgE response or may induce a false-positive IgE response with a non-allergenic food [27]. Viewed through this lens, the process of sensitization in our model mimicked that in human subjects.

Female BN rats have statistically significantly higher specific IgE titers and number of responders than males irrespective of age [27]. Thus, male BN rats were chosen to exclude the influence of gender in our study. As presented in our research, during the sensitization period, all rats appeared healthy with similar weight and length between the hypersensitivity group and control group. The hypersensitivity group remained constant in weight and length the course of the study, in agreement with Ganeshan et al.'s research in mice [28]. Similar findings were observed by Abril and his colleagues [29], who established a food allergy by using an adjuvant in BN rats on their 42-day research. Additionally, no severe symptom, such as death, was observed. In the hypersensitivity group two rats presented a score of 1, eleven had a score of 2, and two had a score of 3; no abnormal performance was found in the control group. This may indicate that the model is mildly ovalbumin-allergic, and that mild food allergy has little effect on long-term growth and development, even though it causes mild intestinal inflammation.

The activation of T cells to the Th2 phenotype is a critical step in the immune mechanisms of food allergy [30]. Th1 cells release type 1 cytokine IFN-γ, whereas Th2 cells release IL-4 and IL-5. These cytokines play key roles in the production of antigen-specific IgE by B cells, which could lead to an increased number of mastocytes [31]. Mast cells are major effectors in food allergy, and their levels in intestinal mucosa correlate with the severity of symptoms in food allergic model [32, 33]. Eosinophils, by releasing various toxic granule proteins, lipid mediators and proinflammatory cytokines, are another principal effector cell type that induces gastrointestinal tissue injury and disease pathogenesis [34]. In our study, Th2-related cytokines such as IL-4 and IL-5 were upregulated, whereas Th1-related cytokine IFN-γ was lower/normal in the hypersensitivity group compared with the control group. In addition, mast cells and eosinophils were markedly increased in the hypersensitivity group, and the levels of ovalbumin-IgE and histamine were higher than in the control group. No adjuvant was used in the experiment, so we cannot determine whether there was immunologic tolerance. This might cause the mild reactions in the final challenge for the hypersensitivity group. Above all, this is a mild reaction allergic model mediated by ovalbumin-specific IgE.

The imbalance of Th1/Th2 cells and the hyperactive Th2 response are the key events of food allergy. The initial stage of food allergy is the activation of T cells, pushing the differentiation of CD4^+^ T cells into Th2 cells in Payer's patches, mesenteric lymph nodes, and spleen. In this step, mature dendritic cells and macrophages present processed antigen to and activate T cells. The maturation of DC requires a signal mediated by Toll-like receptors, especially TLR4 [35, 36]. When the antigen appears, it is displayed by an antigen-presenting cell such as DC presenting TLR, resulting in the activation of CD4^+^ T cells [37, 38]. S100A8/A9 is mainly distributed in neutrophil granulocytes. In the processing of food allergens, eosinophil and neutrophil granulocytes were activated, leading to increased S100A8/A9. Of note, S100A8/A9 is primarily characterized in regard to neutrophils and macrophages as a component of the innate immune system, peculiarly given the reports that its secreted complex is a direct ligand for TLR4 and a modulator of dendritic cell differentiation [8, 39]. The mRNA level of TLR4 dramatically increased in both liver and jejunum, as Westerholm et al. [40] had found in allergic children's intestinal mucosa. Similar reports have demonstrated that S100A8/A9 is both the endogenous ligand [41] and exogenous ligand [7] of TLR4. Additionally, Cheng et al. [8] found that S100A8/A9 enhances endotoxin and initiates the inflammatory response by directly activating TLR4 [7]. Calprotectin, as an inflammatory factor, when combined with TLR4, activates a number of signal transduction pathways, including extracellular regulated kinase, p38 mitogen-activated protein kinase, and protein kinase C. Finally, in the coordination of myeloid differentiation factor 88 [42], calprotectin activates NF-κB [43] and promotes the production of downstream inflammatory cytokines, such as IL-6, IL-1β, and TNF-α. As expected rats in this model were primed, leading to a higher level of ovalbumin-IgE and calprotectin after challenge with a high dose of ovalbumin. Ovalbumin-IgE, fecal calprotectin, and S100A8/A9 in plasma exhibited higher levels in the hypersensitivity group. The expression levels of S100A8, S100A9, and S100A8/A9 in jejunum were increased over time in the hypersensitivity group. The levels of inflammation-associated cytokines, such as TLR4, TNF-α, NF-κB, IL-1β, and IL-6 were also increased in hypersensitivity group. There were positive correlations between TLR4, NF-κB, and TNF-α with S100A8/A9 in feces. Fecal calprotectin was positively correlated with S100A8/A9 in plasma, as was reported by Roseth [44], and with TLR4, TNF, and NF- kappa B. It is not difficult to speculate that elevated S100A8/A9 regulates the balance of Th1/ Th1 cells through the TLR4 pathway and amplifies the cascade of allergic factors and inflammatory factors in food allergy.

*In vitro* cell research, using DC-related cells, such as CD11c^+^, CD86^+^, and MHC II^+^ showed an increasing trend from the control group to ovalbumin group, combined S100A8+ovalbumin group, S100A9+ovalbumin group, and S100 A8/A9+ovalbumin group. Among these five groups, we found that both the S100A8+ovalbumin and S100A9+ovalbumin groups had reduced CD11c^+^, CD86^+^, and MHC II^+^ compared to S100 A8/A9+ovalbumin, This illustrated the function of S100A8/A9 acting on DC. The result also agrees with a study by Yano [45], which found that S100A9^−/−^ gene knockout rats expressed normal mRNA S100A8, but did not express S100A8 and S100A9 protein. These observations may explain the varying proportions of DC-related cells among different groups. S100A8, S100A9, and S100A8/A9 are damage-associated molecular pattern molecules [46], which are highly elevated in patients suffering from atopic dermatitis [47–49] and can be detected in some inflammatory reactions in activated keratinocytes [50]. Moreover, DC and CD4^+^ co-culture solution had higher levels of IL-4 and IL-5 in the four experimental groups, especially in the S100A8/A9+OVA groups, than in the control group. It is not difficult to speculate that besides being an inflammatory factor, S100A8/A9 has some role in the process of a food allergy, possibly as a trigger that amplifies cascade reaction of allergic-related and inflammatory factors in allergic processing. Cheng and colleagues previously reported [8] that S100A8/A9 amplifies the endotoxin-triggered inflammatory responses of phagocytes by directly activating TLR4 [7]. Calprotectin combined with TLR4 activates different signaling pathways, including extracellular regulated kinase (ERK), p38 mitogen-activated protein kinase (p38 MAPK), and protein kinase C (PKC), especially under the synergistic effect of myeloid differentiation factor 88 [42]. NF-κB was activated [43], which promotes the production of downstream inflammatory factors, such as IL-6, IL-1β, and TNF-α.

Establishing a food allergy model without immune adjuvant is closer to the human food allergy process, reduces the distress to the experiment animals, and is consistent with animal ethics [51]. Our research report followed the Animal Research Reporting *In Vivo* Experiments Guidelines (ARRIVEG) [52] published by the international laboratory animal 3R center.

This study found that S100A8/A9 is a key contributor in promoting food allergy development. In addition, we found that fecal calprotectin may be used to predict food allergy in children. Still, this finding has limitations. Only male BN rats were selected in our study, which inevitably caused gender bias. Moreover, though the animal disease model was similar to human disease to some extent, it still could not completely represent a series of pathological changes in human disease, so the results provided by this animal study may not fully represent the situation of human. Third, while calprotectin is obviously elevated in allergic rats, the mechanism by which calprotectin regulates the allergic inflammation requires further research. Last but not least, some specific questions are not addressed in this animal model. First, the level of fecal calprotectin in children younger than 4-year-old, especially infant in the first year of life had several cut-off levels [53, 54], which are not addressed by this animal model. Second, a group for ovalbumin washout period was not included. Thus, the difference in the level of calprotectin in allergic rats with or without washout periods can not be checked, which is another future experiment.

## MATERIALS AND METHODS

### Animals

Eighty 3-week-old male Brown Norway (BN) rats, SPF grade, obtained from the Animal Center of the Chinese Academy of Medical Sciences (Shanghai, China) were maintained on ovalbumin-free rodent diet under specific pathogen-free conditions in accordance with standard guidelines for the care and use of animals [55]. All of the rats were allowed to adapt to the animal house conditions for 4 days with a light-dark cycle of 12 hours at constant temperature (23 ± 3)°C and a relative humidity of 50–70%.

The 80 male BN rats were divided into two groups by random number table, with 40 rats in each group kept on ovalbumin-free rodent diet for 42 days. Every 7 days after intervention with ovalbumin (hypersensitivity group) or NS (control group), 5 rats in each group were sacrificed by cervical dislocation and given a burial after tissue dissection.

### Antigen sensitization and challenge

Rats in the hypersensitivity group were orally gavaged with 1 mg ovalbumin (1 mg ml^−1^ per rat) (Sigma-V, purity > 95%) daily for 41 days, using a 12-gauge stainless steel animal feeding needle. During this stage, the control group was sham-sensitized with normal saline (NS). On day 42, each rat in the hypersensitivity group was orally challenged with 100 mg ovalbumin; while rats in the control group were given an equal volume of NS. Immune adjuvant was unused during the whole molding time. Time for gavages and the measurement of weight and length were fixed every morning. Weight and length were determined each day. On day 42 after challenge by high dose ovalbumin (100 mg), symptom scoring was performed, and feces was collected from rats of each group. Five BN rats on day 7, 14, 21, 28, and 35; and fifteen rats on day 42 in each group were killed by cervical dislocation. Meanwhile, feces was collected before anesthesia; blood was collected after anesthesia, and tissue samples including liver, spleen, and jejunum were collected for further analysis after rats were sacrificed. The observers and statisticians were blinded to the condition of each animal.

This random control experiment was approved by the Institutional Ethics Committee of Xinhua Hospital Affiliated to Shanghai Jiao Tong University (Approval No. XHEC-F-2015-032). All experimental procedures were carried out by the guidelines of the Shanghai Experimental Animal Society, China. Our study has not been registered on any publicly accessible database.

### Cell culture

Thigh and shin bones of 5 BN rats in the control group were taken to isolate bone marrow-derived dendritic cells (DC). CD4^+^ T cells were collected from the spleens of 5 rats in the hypersensitivity group. The thigh and shin bones were taken from BN rats in a bacteria-free operating environment. According to the relevant operating procedures to culture bone marrow derived-dendritic cells (DC), the purity of DC were detected by flow cytometry (FAC). The spleens of BN rats were separated, and CD4^+^ T cells were cultured. DC and CD4^+^ T cells were cultured separately. On the fifth day, DC were divided into five groups: (1) ovalbumin group, which received 20 μg ml^−1^ ovalbumin; (2) combination of S100A8+ovalbumin groups, which receieved 1 μg ml^-1^ S100A8 and 20 μg ml^−1^ ovalbumin; (3) combination of S100A9+ovalbumin groups, which receieved 1 μg ml^-1^ S100A9 and 20 μg ml^−1^ ovalbumin; (4) recombination of S100A8/A9+ovalbumin groups, which received 1 μg ml^−1^ S100A8/A9 and 20 μg ml^−1^ ovalbumin; and (5) control group. After 48 hours, the supernatants of the five groups were collected and the DC surface molecules CD86, CD11c, and MHC II were detected by FAC; the suspension and semi-adherent cells were collected and centrifuged. RPMI 1640 was used to wash cells 3 times and to adjust the cell concentration of each group to 2 × 10^5^ ml^−1^. CD4^+^ T cells were collected and adjusted to a cell concentration of 1 × 10^6^ ml^−1^. We took 0.1 ml of cells from the 5 groups of DC into 96-well plates and added 0.1 ml CD4^+^ T cells for mixed culture. Each concentration was plated in triplicate and cultured for 48 hours at 37 C and 5% CO_2_. The supernatant was collected to determine the levels of IL-4, IL-5, and INF-γ by ELISA.

### Measurement of serum immunoglobulin level

Angular venous blood was obtained weekly after initial sensitization to monitor levels of serum ovalbumin-IgE antibody responses by ELISA. Sera were collected and stored at −80°C. The level of ovalbumin-IgE was used to determine success of molding [17]. All steps followed the protocol described by the manufacturer (sbjbio, Inc., Nanjing, Jiangsu, China). All analyses were performed in duplicate, and coefficients of variation of greater than 10% were repeated to ensure a high degree of precision.

### Assessment of hypersensitivity reactions

Anaphylactic symptoms were evaluated within 30 minutes after high dose ovalbumin challenge, and a scoring system was used (Table 2) [56].

**Table 2:** Scoring system to evaluate systemic hypersensitivity reactions [56]

Score	Symptoms
0	No symptoms;
1	Scratching and rubbing around the nose and head;
2	Puffiness around the eyes and mouth, diarrhea, polar erection, reduced activity, and/or decreased activity with increased respiratory rate;
3	Wheezing, labored respiration, and cyanosis around the mouth and the tail;
4	No activity after prodding or tremor and convulsion;
5	Death.

Symptom scoring was independently performed by two investigators in a blind method.

### Measurement of plasma histamine levels

To determine plasma histamine levels, blood was collected 30 minutes after intragastric gavage sensitization or challenge on day 7, 14, 21, 28, 35, and 42. Plasma was prepared and stored at –80°C until analyzed. An enzyme immunoassay kit (sbjbio, Inc., Nanjing, Jiangsu, China) was used to measure the levels of histamine, according to the manufacturer's methodology.

### Measurement of fecal calprotectin

Feces from each rat of the two groups was collected after intragastric gavage sensitization or challenge on day 7, 14, 21, 28, 35, and 42. Fecal samples were stored at –80°C until analyzed. An enzyme immunoassay kit (sbjbio, Inc., Nanjing, Jiangsu, China) was used to measure the levels of fecal calprotectin, according to the manufacturer's methodology.

### Cytokine measurement

100 mg of spleen was collected. Tissue was homogenized at 1200 rpm for 15 min to extract the supernatant. According to the manufacturer's instruction, ELISA testing was performed to detect INF-γ, IL-4, IL-5. Each sample was measured three times, and the average value was taken.

### Histology and morphometry

Jejunum tissues were collected, fixed in 4% paraformaldehyde, and embedded in paraffin, then cut longitudinally into 5 μm thick sections and processed for hematoxylin and eosin (H&E staining) and toluidine blue staining or immunohistochemistry. Mast cell activation status was determined by counting cells with dense metachromatic granules and compact shape versus those with dispersed granules extending clearly outside the cell body. Immunohistochemistry was performed using standard protocols with the following antibodies: IL-1β, (1:100, both from Origene, Inc., Rockville, MD, USA); TNF-α, S100A8, S100A9 (1:200, Abcam, Inc., Cambridge, MA, USA); Toll-like receptor 4 (TLR4), S100A8/A9 (1:200, NOVUSbio, Inc., Littleton, CO, USA); nuclear factor kappa B (NF-κB) (1:200, Cell Signaling Technology, Inc., Danvers, MA, USA).

### RNA isolation and real-time PCR

The total RNA of cells was isolated using Trizol reagent (Invitrogen, Carlsbad, CA, USA) according to the manufacturer's instructions. After the reverse transcription reaction, real-time polymerase chain reaction (PCR) was performed with an ABI 7900HT system using SYBR^®^ Premix (Takara, Dalian, China) according to the manufacturer's instructions. The conditions of the real-time PCR were as follows: denaturation at 95°C for 10 s, 40 cycles at 95°C for 10 s, and 60°C for 30 s. A dissociation stage was added to the end of the amplification procedure. No nonspecific amplification was observed, as determined using the dissociation curve. Glyceraldehyde 3-phosphate dehydrogenase (GAPDH) was used as an internal control. The data were analyzed using the comparison Ct (2^−ΔΔCt^) method and expressed as the fold change relative to the respective control. Each sample was analyzed in triplicate. The primer sequences used in this study were as follows: Actin: forward, 5′- TGT GTT GTC CCT GTA TGC CTC TG-3′; reverse, 5′- ATA GAT GGG CAC AGT GTG GGT G-3′; IL-6: forward, 5′- ATA TGT TCT CAG GGA GAT CTT GGA A-3′; reverse, 5′- GTG CAT CAT CGC TGT TCA TAC A-3′; IL-1β: forward, 5′- CAA ACA ATA CCC AAA GAA GAA G-3′; reverse, 5′- AGT CAA CTA TGT CCC GAC CA-3′; NF-κB: forward, 5′- ACA GAG GAA ACG CCA GAA GC-3′; reverse, 5′- AAT GCA ATC CCA CCG TAA GC-3′; TNF-α: forward, 5′- GTG ATC GGT CCC AAC AAG GA-3′; reverse, 5′- AGG GTC TGG GCC ATG GAA-3′ ; TLR4: forward, 5′- GGC ATC ATC TTC ATT GTC CTT G-3′; reverse, 5′- AGC ATT GTC CTC CCA CTC G-3; S100A9: forward, 5′- TCA TGG AGG ACC TGG ACA CAA A-3′; reverse, 5′- GCA GCT TCT CAT GAC AGG CAA A-3; S100A8: forward, 5′-ACT GGA GAA GGC CTT GAG CAA C-3′; reverse, 5′- ATC CCT GTA GAG GGC ATG GTG A-3; IL-4: forward, 5′-CAA CAA GGA ACA CCA CGG AGA A-3′; reverse, 5′- AAG CAC GGA GGT ACA TCA CG-3; IL-5: forward, 5′-GAG GCT TCC TGT TCC TAC TC-3′; reverse, 5′- CTC GGA CAG TTT GAT TCT TC-3; INF-γ: forward, 5′-TGT CAT CGA ATC GCA CCT GA-3′; reverse, 5′- TGT GCT GGA TCT GTG GGT TG-3.

### Statistical analyses

The SPSS software package (version 16.0 for Windows; SPSS, Inc., Chicago, IL, USA) was applied for statistical analysis. All values are presented as means ± S.E. Student's *t* test. Mann-Whitney *U* tests were used for comparison between different groups. One-way analysis of variance was conducted in multiple-group testing. A simple regression analysis was carried out to estimate the correlations of S100A8/A9, TLR4, NF-κB, or TNF-α with fecal calprotectin, the relationship between whose values were performed by Spearman's correlation test. All of the tests were 2-sided, and Probability (*P*-value) of < 0.05 was supposed to be significant.
